# Differences in Distribution of Potassium-Solubilizing Bacteria in Forest and Plantation Soils in Myanmar

**DOI:** 10.3390/ijerph16050700

**Published:** 2019-02-27

**Authors:** Xin Dong, Le Lv, Weijun Wang, Yongzhi Liu, Chunhua Yin, Qianqian Xu, Hai Yan, Jinxia Fu, Xiaolu Liu

**Affiliations:** 1 School of Chemistry and Biological Engineering, University of Science and Technology Beijing, Beijing 100083, China; dxzyfx@163.com (X.D.); happylvle@163.com (L.L.); 17888803570@163.com (W.W.); abc5678579@163.com (Y.L.); Chyin@ustb.edu.cn (C.Y.); qianqianxu@ustb.edu.cn (Q.X.); haiyan@ustb.edu.cn (H.Y.); 2 Hawaii Natural Energy Institute, University of Hawaii, Honolulu, HI 96822, USA

**Keywords:** Rubber tree, bacterial community, rhizosphere, potassium solubilizing bacteria, microbial ecology

## Abstract

Potassium (K) has been recognized as an essential element in intensive agricultural production systems, and deficiency of K usually results in a decrease in crop yields. The utilization of potassium-solubilizing bacteria (KSB) to increase the soluble K content in soil has been regarded as a desirable pathway to increase plant yields. Following the inoculation of KSB in the soil, potassium can be released (in the form of K^+^) and consumed by plants. This study aims to investigate and compare the distribution characteristics of potassium-solubilizing bacteria between forest and plantation soils in Myanmar. In this study, 14 KSB strains were isolated from rhizosphere samples collected from forest soil, as well as fertilized rubber tree rhizosphere soil and fertilized bare soil from a plantation. Broadleaf forests with high levels of canopy cover mainly comprised the forest environment, and rubber trees were planted in the plantation environment. The Chao and abundance-based coverage estimator (ACE) indices showed that the microbial abundance of the plantation soil was higher than that of the forest soil. According to the Illumina MiSeq sequencing analysis results, the Shannon index of the forest soil was lower while the Simpson index was higher, which demonstrated that the microbial diversity of the forest soil was higher than that of the plantation soil. Potassium-solubilizing test results showed that the strains E, I, M, and N were the most effective KSB under liquid cultivation conditions. Additionally, KSB only accounted for less than 5.47% of the total bacteria detected in either of the sample types, and the distribution of dominant KSB varied with the soil samples. As another result, the abundance of *Pseudomonas* spp. in S1 was higher than in S2 and S3, indicating a negative impact on the growth of *Pseudomonas* in the fertilized rubber tree rhizosphere soil. The significance of our research is that it proves that the increasing use of KSB for restoring soil is a good way to reduce the use of chemical fertilizers, which could further provide a relatively stable environment for plant growth.

## 1. Introduction

*Hevea brasiliensis* (rubber tree) is the major source of natural rubber and is traditionally cultivated in equatorial countries, humid areas with tropical and monsoon climates [1]. Although rubber trees usually have a 7-year-long immature, unproductive period, the economic lives of rubber trees can reach 15–30 years, or even longer [2]. Chemical fertilizers are typically used for sustaining the yield, following long-term intensive rubber tapping in plantations. Potassium (K) is one of the most essential elements for plant growing, and K-deficient soils are often acidic, sandy, saline, and waterlogged [3]. K has been recognized as an essential element in intensive agricultural production systems, especially in organic soils [4]. A K deficiency usually results in both a decrease in crop yields and resistance to pathogens and insect pests [5,6].

In general, soils host a large variety of microbial communities [7,8]. Most edaphic microbes, however, cannot be cultured under laboratorial conditions, which restrains investigation related to the influence of human activity on specific changes in the soil bacterial community structure [9]. Previous studies showed that high-throughput sequencing can assist in obtaining overall assessments of bacterial diversity, community structure, and the relative abundances of specific bacterial taxa in soil [10,11,12]. Potassium reserves in the crust are usually very high, accounting for 2.1% to 2.3% of the crust, but only 1–2% of potassium is water soluble and/or exchangeable in soil [13]. Although most K existing in soil is in the form of a mineral, this K can be solubilized and absorbed by microbes and plant roots when different kinds of KSB are inoculated into a soil or fluid medium under laboratory conditions [14,15,16].

The proportion of potassium-dissolving bacteria in the flora environment has not been well obtained from traditional methods, and high-throughput 16S rDNA sequencing provides a way for us to analyze the microbial community structure. Comparing the screened KSB with the sample flora, we can understand the content of KSB in the flora. Potassium-solubilizing bacteria (KSB) isolated from the rhizosphere of the plant can improve the K transition. Three types of KSB (i.e., *Bacillus* spp., *Enterobacter* spp., and *Pseudomonas* spp.) have been discovered and characterized [14,17,18,19,20]. These KSB can rapidly release K from K-bearing minerals, though their organic acid secretions [21]. These organic acids can either directly dissolve rock K (K-containing minerals) or chelate mineral silicon ions to release K into solution, such as indole-3-acetic acid released by *Enterobacter* sp. DMKU-RP206 [22,23]. It has been reported that KSB inoculation in soil has positive effects, under controlled conditions, in cucumber, eggplant, peanut, and pepper plantations [14,24]. Thus, the use of KSB as a biological fertilizer has gained considerable attention recently.

The utilization of KSB to increase the soluble K content in soil has been regarded as a desirable pathway to increase plant yield [19]. A paucity of studies, however, have been conducted on rubber tree rhizosphere soil and its relative abundances. In the present work, the soil bacterial diversity and community structure in plantations were investigated and compared with that of forests in the same region. The KSB were isolated from the rubber tree rhizosphere soil and tested for their capacity for K release. In addition, the community structure of KSB, replicated from plantation soil, was also studied and compared with that from forest soil.

## 2. Materials and Methods

### 2.1. Samples and Elemental Analysis

Soil samples were aseptically collected from a forest and a rubber tree plantation in Myanmar. All samples were collected from the layer 5–25 cm in depth, from soil at three different coordinates. The soil samples were collected from three regions: 23°45’ N, 96°12’ E (S1); 24°13’ N, 96°40’ E (S2); and 24°30’ N, 96°32’ E (S3). The soil collected in S1, S2, and S3 was forest soil (from mainly broadleaf forests with high levels of canopy cover), fertilized rubber tree rhizosphere soil from a rubber tree plantation, and fertilized bare soil from a plantation, respectively. Soil samples were collected in triplicate at each test site. The soil samples were sealed in sterile Ziploc bags to minimize any possible exogenous microbial contamination. Part of the mixed samples was stored at −20 °C, for future analysis. Soil pH was assessed using the saturated paste method with a pH meter. A 10 g soil sample was placed in the bottle and then 25 mL of CO_2_-free water was added. After shaking for 30 min and standing for 30 min, it was measured with a pH meter (INESA Scientific Instrument Co. Ltd., Shanghai, China). The soil samples were delivered to the testing center at the University of Science and Technology Beijing (Beijing, China), to test the concentration of K^+^ (potassium ions), Mg^2+^ (magnesium ions), Si (silicon), P (phosphorus), and total N (nitrogen). The following procedure was followed: First, take 5 g of soil sample in the bottle; second, add 50 mL of deionized water; third, shake for 30 min; finally, filter with double-layer filter paper and 0.45 μm filter (AIJIREN, Zhejiang, China). A Dionex DX-120 ion chromatograph (Thermo Fisher Scientific, Waltham, MA, USA) with CS12A cation separation column and conductivity detector was employed to determine the K^+^ and Mg^2+^ content of the soil. The Kjeldahl method, Molybdate colorimetric analysis, and Molybdenum blue colorimetric analysis were used to analyze the N and P content. The carbon content of the soil was analyzed using the traditional method of potassium dichromate oxidation–external heating [25].

### 2.2. DNA Isolation and Illumina MiSeq Sequencing

A total of 0.1 g of soil from each sample was used for the isolation of soil DNA. We normalized isolated samples to a concentration of 10 ng/μL. Genomic DNA was extracted by using a FastDNA SPIN KIT for Soil (MP Biomedicals, LLC, California, USA, Catalog: 116560200), and a pair of primers (515F/907R) were used for the PCR of the 16 S rRNA gene V4 region. These samples were barcoded separately, to enable multiplex sequencing. The PCR reactions were conducted in a 20 μL master mix with 4 μL 5x FastPfu Buffer, 2 μL dNTPs (2.5 mM), 0.4 μL forward primer (5 μM), 0.4 μL reverse primer (5 μM), 0.4 μL FastPfu polymerase (TransGen AP221-02), and 10 ng template DNA. The amplification process was conducted according to the following procedure: (1) Five min initial denaturation at 94 °C; (2) 11 cycles of denaturing at 95 °C; (3) annealing at 60 °C for 30 s; (4) extension at 72 °C for 1 min; (5) 25 cycles of denaturing at 95 °C; (6) annealing at 50 °C for 30 s; (7) extension at 72 °C for 1 min; and (8) extension at 72 °C for 10 min. DNA marked with different barcodes were sequenced by the Illumina MiSeq platform (Shanghai Majorbio Bio-pharm Technology Co., Ltd., Shanghai, China).

### 2.3. Isolation of KSB from Soil Samples in the Laboratory

A 1 g soil sample was added into 100 mL sterile saline solution (0.8% NaCl) and mixed thoroughly by shaking the flask on a rotatory shaker for 5 min at room temperature. Next, 1 mL soil solution was extracted in 9 mL sterile saline solution and centrifuged for 30 s. After being diluted 7 times, 0.1 mL of each degree suspension was spread over resterilized selective agar plates in triplicate. The selective medium was prepared with 10 g sucrose, 0.5 g yeast extract, 1 g (NH_4_)_2_SO_4_, 2 g Na_2_HPO_4_, 0.5 g MgSO_4_·7H_2_O, 1 g CaCO_3_, 1 g potash feldspar (9.60% K_2_O), 15 g agar, and 1 L sterile water [26]. The inoculated plates were incubated at 29 ± 1 °C for 48 h. Fast-growing and slime colonies were selected and purified on the selective medium. Colonies with a transparent zone after 5 days incubation were KSB. Gram staining was performed, as previously described [27]. The microbial biomass of each strain was grown in a beef extract peptone culture medium. Single colonies were placed in liquid lysogeny broth (LB) medium to be cultured, and the bacterial suspension was stored at −20 °C in glycerin, for later use.

### 2.4. Amplified Ribosomal DNA Restriction Analysis (ARDRA)

Chromosomal DNA was extracted by using a rapid bacterial genomic DNA isolation kit, according to the following procedure: (1) Centrifuge 1 mL bacterium culture solution using a 1.5 mL microfuge tube at 8000 rpm for 1 min at room temperature; (2) remove the clear supernatant; (3) resuspend the bacteria by adding 180 µL lysozyme solution (20 mg/mL) and storing at 37 °C for 1 h; (4) mix the solution with 200 µL buffer PB and store at −20 °C; (5) centrifuge the solution at 10,000 rpm for 5 min at room temperature and place the clear supernatant into a 1.5 mL microfuge tube; (6) add isovolumetric isopropanol to the microfuge tube, store at room temperature for 3 min, centrifuge at 10,000 rpm for 5 min, and abandon the clear supernatant; (7) add 1 mL 75% ethyl alcohol into the tube, mix for 2 min, centrifuge at 10,000 rpm for 2 min, and abandon the clear supernatant; (8) repeat step 7, open the lid, and sit for 10 min at room temperature; (9) dissolve the DNA by using 50 μL TE buffer solution in the tube and rinse for 2 min, then centrifuge at 10,000 rpm for 2 min, abandon the clear supernatant, repeat step 5 and step 7, open the lid and invert for 10 min at room temperature. Repeat step 8, and then conduct PCR amplification of 16S rDNA as previously reported [28]. A pair of universal primers (27F/1492R) was used to amplify the 16S rDNA conserved sequence [29]. After the amplification process, the PCR product (10 μL) was digested with the restriction endonucleases AluI and RsaI (Appligene, France) for 16 h at 37 °C, and BstUI (Ozyme, Saint-Quentin-en-Yvelines, Versailles, France) for 4 h at 60 °C. The obtained products were then separated on 3.5% (w/v) MetaPhor agarose gels in 1 × TAE buffer. The DNA fragments obtained were compared with the DNA molecular mass marker Trans2K (Transgen, Beijing, China), and Gel Doc XR+ System (Bio-Rad) restriction profiles were classified, based on the presence of the DNA fragments for each enzyme. The unweighted pair group method with arithmetic mean (UPGMA) was used to build the similarity dendrogram from the distance matrix, using the NTSYS software (Exeter Software, Setauket, NY, USA) [30].

### 2.5. Sequencing and Analysis of Data

The 16S rDNA was amplified with Pfu DNA polymerase, cloned into pGEM-T Easy Vectors, and transformed into *Escherichia coli* DH-5α competent cells, according to the manufacturer’s instructions. The lysates were used as DNA templates in the subsequent PCR amplification, and the transformants were checked by agarose gel electrophoresis. Ambiguities were resolved or confirmed by repeated sequencing. Three isolates were picked and purified for sequencing from each of the types. The sequences determined in this study were aligned with the 16S rRNA sequences retrieved from the GeneBank database. These 16S rDNA sequences represented all of the isolate sequences obtained from each soil sample. The 10 sequences were compared to the GeneBank entries by using the Basic Local Alignments Search Tool (BLAST) of The National Center for Biotechnology Information (NCBI) to obtain a preliminary phylogenetic affiliation of the isolates, and closely related strains with our isolates were integrated to construct a phylogenetic tree. The 16S rRNA gene sequences were aligned using the Clustal X 2.0 program (EMBL-EBI, Hinxton, UK), and the phylogenetic tree was constructed using the neighbor-joining (NJ) algorithm with MEGA 6.06 (http://www.megasoftware.net) [31].

### 2.6. Determination of Potassium-Solubilizing Capability

The measurements of capacity of K release were done in triplicate, to investigate the 14 isolated KSB from the soil samples. First, 100 mg potash feldspar was mixed with 50 mL nutrient medium, and the pH value of the solution was adjusted to 7.0 before steam sterilization. After continuous enrichment and culturing twice, 100 μL of bacteria suspension was inoculated into 10 mL sterilized culture media, and the culture media (without inoculation) was set as a control. After culturing at 200 rpm for 5 days at 35 °C, the bacteria suspension was treated with 6% H_2_O_2_ at 95 °C, and then filtered to collect the mineral powder residues from the cells and media. The filtrate solutions were digested with 30% H_2_O_2_ in a water bath at 95 °C, and then diluted to 50 mL with distilled water. The potassium concentrations in the solution were measured by an atomic absorption spectrophotometer (AAS; Shimadzu, AA6800, Kyoto, Japan).

## 3. Results

### 3.1. Characteristics of Soil Samples

The chemical characteristics of the three samples are listed in Table 1. The concentration of N was at least two orders of magnitude higher than that of the other elements (concentrations of N were 5.366, 3.805, and 5.476 mg/g for S1, S2, and S3, respectively). The N content in S1 and S3 was similar, but was significantly higher than that of S2. As the contents of K, Mg^2+^, Si, and P varied with the soil samples, the characteristics of the soil samples were also varied. For instance, the concentrations of soluble K and P in S2 and S3 were significantly lower than in S1 (Table 1). The pH values of the S1, S2, and S3 samples were 6.24, 6.47, and 6.62, respectively. The organic carbon soil contents (SOC) in the S1, S2, and S3 samples were 2.6964%, 3.0071%, and 2.5452%, respectively.

### 3.2. Enumeration of Total Bacteria

A total of 149 isolates capable of potassium-release were identified, out of the total of 1678 isolated bacteria. The percentages of potassium-solubilizing bacteria (KSB) of the total amount of aerobic bacteria isolated were 12.25% (80 isolates out of 653) for S1, 5.46% (29 isolates out of 531) for S2, and 8.10% (40 isolates out of 494) for S3 (see Table 2). A total of 80, 29, and 40 clone sequences were included in phylogenetic analysis, which generated 8, 7, and 9 operational taxonomic units (OTUs), respectively.

### 3.3. Bacterial Abundance and Diversity

**Figure 1:**
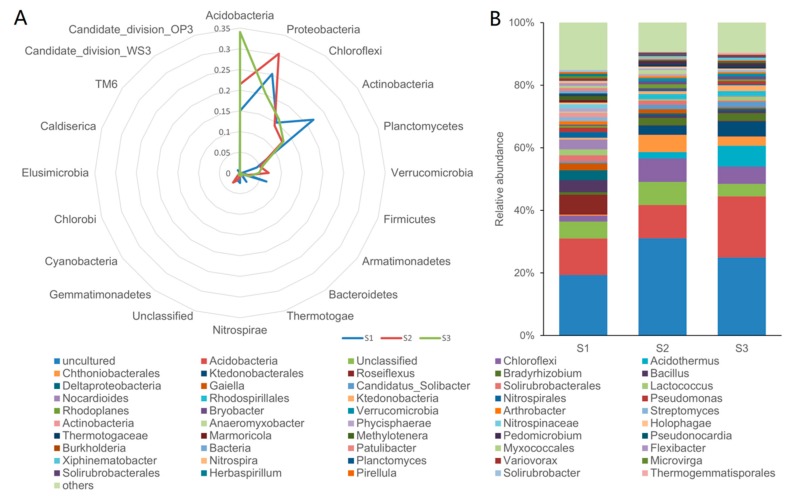
(**A**) Taxonomic distribution of soil samples, phylum distribution of all samples; (**B**) genus distribution of all samples. S1: forest soil; S2: fertilized rubber tree rhizosphere soil from a rubber tree plantation; S3: fertilized bare soil from a plantation.

A total of 58,388 quality sequences, obtained from soil samples, were assigned a taxonomic classification with an average length of 397 bp. Table 3 lists the OTUs of the three soil samples. The number of OTUs (97% similarity) ranged from 702 to 803, and the coverage estimates ranged from 96.8–98.0%, indicating that almost all of the bacterial species were included in the analysis. The Chao and abundance-based coverage estimator (ACE) indices showed that the microbial abundance of the plantation soil was higher than that of the forest soil. According to the Illumina MiSeq sequencing analysis results, the Shannon index of the forest soil was lower while the Simpson index was higher, which demonstrated that the microbial diversity of the forest soil was higher than that of the plantation soil. The relative abundance level of the top 20 taxonomic class categories is summarized in Figure 1A. *Proteobacteria, Actinobacteria, Acidobacteria*, and *Chloroflexi* were the most dominant four phyla in S1, as well as in S2 and S3. The abundance of *Proteobacteria* in the forest and fertilized rubber tree rhizosphere soils (S1 and S2; 25.25% and 30.30%, respectively) was significantly higher than in the fertilized bare soil from a plantation (S3; 20.12%). As the abundance of *Acidobacteria* in the S1, S2, and S3 samples was 15.04%, 21.43%, and 34.09%, respectively, the ratio between *Proteobacteria* and *Acidobacteria* was 1.68, 1.41, and 0.59, respectively. Thus, the S1 samples contained more organic matter, which is consistent with the element analysis results. The abundance of *Verrucomicrobia* was low in S1 (0.47%), in comparison with S2 and S3 (6.92% and 4.91%, respectively), whereas the abundance of *Bacteroidetes* and *Nitrospirae* in S1 (2.64% and 2.44%, respectively) was much higher compared to S2 and S3 (both < 0.7%).

### 3.4. Amplified Ribosomal DNA Restriction Analysis (ARDRA) and 16S rDNA Sequencing Analysis

**Figure 2:**
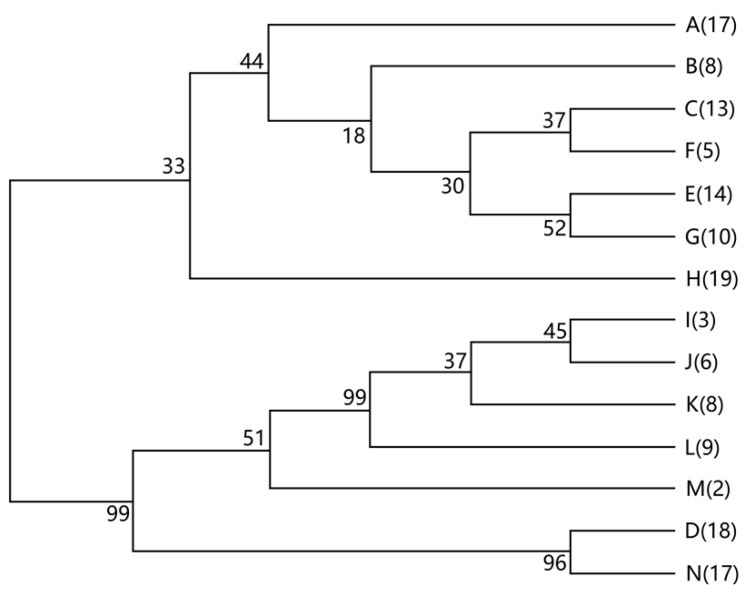
Dendrogram of different denitrifying isolate types. The dendrogram was based on 16S Amplified Ribosomal DNA Restriction Analysis (ARDRA) patterns obtained from soils S1, S2, and S3. The letter indicates the ARDRA type and the numbers in parentheses are the numbers of isolates for each type.

KSB were isolated from three soil samples and all isolates were in a band range of 1053–1075 bp, after PCR amplification with the universal primers. KSB isolates were divided into 14 ARDRA types, according to the similarities of their restriction patterns (see Figure 2). The KSB were identified by the 16S rDNA sequencing GenBank with accession numbers as follows. A: KP974262, B: KP974263, C: KP974264, D: KP974265, E: KP974266, F: KP974267, G: KP974268, H: KP974269, I: KP974270, J: KP974271, K: KP974272, L: KP974273, M: KP974274, and N: KP974275. The number of individual isolates found in each ARDRA type was distinguishable (Table 4). The dominant ARDRA type in soil samples varied, as the type of KSB differed in the soil samples. A, B, C, D, E, G, and L strains were isolated from S1, whereas D, F, G, H, I, J, and K were obtained from S2 and C, D, F, H, J, K, L, M, and N strains were isolated from S3 (Table 4). The relative abundance of these ARDRA types also varied with the soil type and the number of isolates in S1, S2, and S3 was 70, 29, and 40, respectively.

**Figure 3:**
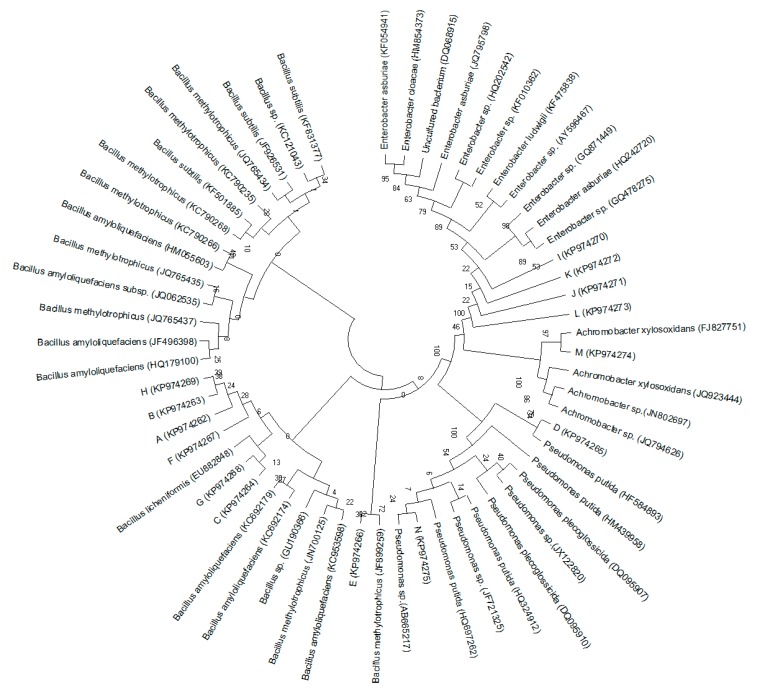
Phylogenetic trees based on the 16S rDNA sequencing, drawn by MEGA 6.06.

Monoclonal bacteria from each strain were selected for the 16S rDNA sequencing analysis. Figure 3 shows the phylogenetic trees generated from the sequences of these strains and their high consensus sequences. These strains can be clustered into four major genera—(I) *Bacillus*: A, B, C, E, F, G, and H [32]; (II) *Enterobacter*: I, J, K, and L; (III) *Pseudomonas*: D and N; and (IV) *Achromobacter*: M. Table 4 lists the number of monoclonal bacteria in KSB that were classified as *Bacillus* in S1, S2, and S3 samples (63, 19, and 4, respectively). A similar trend was also observed in the abundance of *Bacillus*. The abundance of *Bacillus* in S1, S2, and S3 was 3.79%, 0.91%, and 0.76%, respectively, indicating that compound-nutrient forest soil was preferable for the growth of *Bacillus*. Monoclonal bacteria classified as *Pseudomonas* in KSB mainly existed in S1 and S3 (16 and 18, respectively), which was significantly higher than in S2. The abundance of *Pseudomonas* in S1 was also the highest, 1.42% versus 0.23% and 0.92% in S2 and S3, respectively, illustrating that the fertilized rubber tree rhizosphere soil in plantations has a negative impact on the growth of *Pseudomonas*. Furthermore, 26 isolates from KSB were found to be phylogenetically related to *Enterobacter*, which made *Enterobacter* the third most dominant genus (Table 4).

### 3.5. K-Solubilizing Capacity Analysis of KSB Isolates

**Figure 4:**
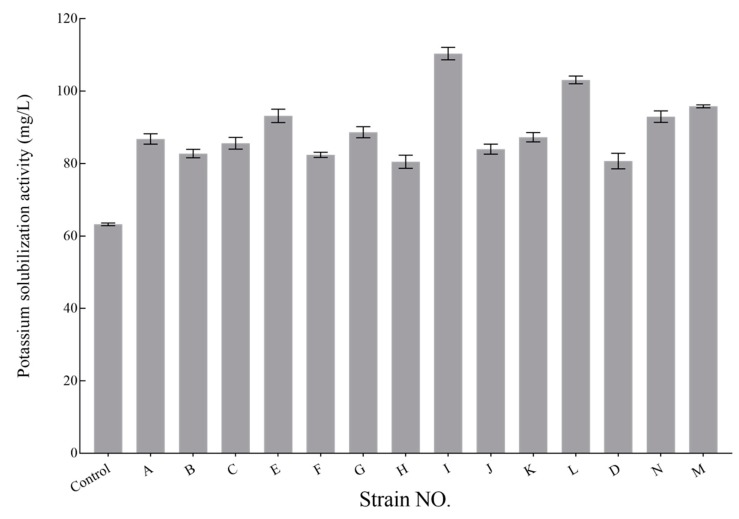
Potassium-solubilizing activity of KSB isolates in aqueous medium. Each bar represents a mean (± standard deviation) value of soluble K content in mg L^−1^. Uninoculated solution was used as a control (first bar). The black lines on the column chart are the marks of the standard error (SE) for three samples from three replications. (*p* < 0.05).

Monoclonal bacteria from each strain were selected to investigate their capacity to release K from potassium feldspar in an aqueous medium, and the efficiency of 14 isolates to solubilize potassium was investigated (Figure 4). The capability of the KSB to solubilize K was in the range of 78.43–109.03 mg/L. Only one isolate displayed low solubilization ability (<80 mg/L). The solubilization capability of 5 isolates was high (>90 mg/L), whereas over 50% of isolates had solubilization capability in the range of 80–90 mg/L. The capacity of all the strains from *Bacillus* was very similar, but significantly lower than the strains I and L from *Enterobacter*. Strain I exhibited the highest capability (109.03 mg/L) (Figure 4). As listed in Table 4, KSB from *Enterobacter* mainly exist in plantation soil, and these KSB showed higher solubilization capability in comparison with those from *Bacillus*.

### 3.6. Impacts of the KSB–Soil Interactions

As shown in Table 3 and Figure 4, the abundance and diversity of KSB decreased after agricultural activities. They all showed different capacities for dissolving K-feldspar in aqueous mediums (Figure 4). The KSB in forest soil are, quantitatively, two to three times more capable than those in plantations, and have a more diverse soil KSB distribution proportion. Although some KSB species of *Enterobacter* did have a better capability (Figure 4), the number of KSB in forest samples was much higher than in the plantation (Table 4), which led to different K^+^ concentrations in the two kinds of samples, to some extent. The soil micro-organism community in the two systems varied considerably (Figure 1), and the richness of KSB in forest soils was much higher, when compared to the plantation soils.

## 4. Discussion

The chemical characteristics of the three soil samples showed that different elements varied with the soil sample origin. Generally, fertilizer selection and amount utilized merely depend on providing an optimal N supply, without considering the K and P content of the soil. Soluble K and P, however, are crucial for plant growth. KSB have been reported to be capable of increasing the soil K content [20]. The content of soluble K in the soil is low when not enough KSB are present. Thus, the lower content of soluble K in plantation soils (S2 and S3) may result from a lack of KSB.

The bacterial abundances, expressed by the Chao index and ACE (richness) index, were significantly different among the three treatments (i.e., S3 > S2 > S1), indicating that the utilization of fertilizer can improve bacterial growth, which is consistent with the results reported by Liu et al. [33]. The soil bacterial diversity of forest soil (S1), however, was greater than that of the plantation soils (S2 and S3), which was reflected in the Shannon and Simpson indices (Table 3), indicating that the long-term utilization of fertilizer can affect the bacterial community composition and structure [33,34,35,36,37]. It has been reported that the bacterial community composition is very sensitive to changes in the soil property; even tree defoliation can change the characteristics of the bacterial community [32,38,39,40]. It is worth noting that the contents of soluble K and P in forest soil (S1) were higher than in plantation soil (S2 and S3), owing to the tree defoliation. Interestingly, the forest soil treatment has greater bacterial diversity in comparison with plantation soil after long-term utilization of fertilizer (S2 and S3). This may result from a balanced nutrient supply, provided by fallen tree leaves. The abundance of *Proteobacteria* in forest and fertilized rubber tree rhizosphere soil was significantly higher than in fertilized bare soil, indicating that the abundance of *Proteobacteria* can be improved by adding leaf litter [41]. It has been reported that the abundance ratio between *Proteobacteria* and *Acidobacteria* is correlated with the trophic status of the soil, and lower ratios were found in oligotrophic environments [42,43]. In general, soils with a higher content of organic matter are more suitable for growth of copiotrophic bacteria, while unfertilized soil is preferred for oligotrophic growth [44,45]. As *Acidobacteria* and *Verrucomicrobia* are both classified as oligotrophic phyla, and their abundances in S1 are relatively low, S1 can be regarded as a copiotrophic soil, which may result from the tree defoliation. The similarity of bacterial community profiles from soils S2 and S3 is high—approximately 70%—in comparison with that of S1 (Figure 1B), indicating that the bacterial community structure in forest soil significantly differs from that in plantation soil, and this may result from the difference in soluble K and P content in the soil samples.

The higher content of KSB in the forest soil may have resulted from extensive rhizospheric colonization, which may have increased the diversity of the bacteria community. The concentration of K^+^ in forest soil is significantly higher than in plantation soil (Table 1), as organic acids secreted by KSB can react with insoluble K and convert it to plant-consumable K^+^ [36], and more organic acids can be secreted when the KSB level is high. More colonies (*Bacillus*) were identified from group I, in comparison with the other groups, demonstrating that *Bacillus* is the dominant group of KSB in all soils investigated. It has been reported that *Bacillus* and *Pseudomonas* are the most abundant strains of KSB isolated from the crop rhizosphere [20]. Inoculation with *Enterobacter* bacterial strains, which had strong activities for solubilizing potassium, was found to facilitate the mobilization of potassium efficiently in plants, when feldspar was added to the soil [46]. Interestingly, 25 *Enterobacter* isolates were found in S2 and S3, in comparison with one isolate in S1, meaning that the growth of *Enterobacter* is affected by the utilization of fertilizer. For *Achromobacter,* limited data was available in comparison with *Bacillus, Enterobacter,* and *Pseudomonas* [47]. In fact, only two monoclonal bacteria isolated in this study can be classified as *Achromobacter*, which further demonstrates that *Achromobacter* is not the major class of KSB.

The K-solubilizing capacity results indicated that all the strains are capable of solubilizing feldspar, but with different efficiencies. It is worth noting that there were many more types of KSB found in forest soil than in plantation soil (80, 29, and 40 types for S1, S2, and S3, respectively). Although the solubilization capability is a critical factor which affects the content of soluble K in soil, the diversity of monoclonal bacteria plays a more important role on solubilizing feldspar. In the forest, most materials will be reused in a long-term cycle. Meanwhile, KSB can continually transform structural K into soluble K, to increase available K for the whole system. However, in the plantation, this cycle has been broken by human agricultural activity. The majority of K supplementation for the plantation came from artificial fertilization. It is not difficult to speculate that the different contents of solution K in forest and plantation soils were largely due to the fertilization treatments, applied to raise economic efficiency. Chemical fertilization and green manure can directly add soluble K and change the soil microbial community [48]. In partial support of our hypothesis, the differences between the forest system and plantation system may be determined by different plant species, along with agricultural and microbial factors [49]. Forests have abundant plant resources and a complete ecological cycle. Richness of plant species and plant functional diversity have a positive influence on the overall catabolic activity and catabolic diversity of the bacterial community [49]. A previous study indicated that plant species differ in their capacity to release mineral K from the soil [50,51,52]. Agricultural activities promote singularizing plant species, and tapped rubber trees in plantation uptake massive quantities of major elements from the soil. Consequently, additional fertilization supplementation cannot maintain dissolved K at a normal level. The effect of agricultural activity is one of the causes of the K deficiency in the plantation. In a plantation system, the K cycle was unbridgeable for its economic role. Therefore, the increasing use of KSB for restoring the soil is a good way to offset the use of chemical fertilizer.

## 5. Conclusions

Potassium-solubilizing bacteria (KSB) from forest and plantation soils were isolated to investigate their capabilities to convert K from feldspar. Fourteen types of KSB strains were successfully identified in the soil samples collected. The results, expressed by the Chao and ACE indices, showed that the microbial abundance of samples in the plantation soils was higher than in the forest soil. The Illumina MiSeq sequencing analysis results indicated that the microbial diversity of samples from the forest soil was higher than that in the plantation soils. These results illustrate that the diversity of micro-organisms and their capability to solubilize potassium from potassium feldspar can be highly affected by human activities. Through the ARDRA analysis, strains E, I, M, and N were found to be the most effective KSB under liquid cultivation conditions. The diversity of monoclonal bacteria plays an important role in solubilizing feldspar, in comparison with the solubilization capability of KSB. As the utilization of fertilizer in plantations can significantly change the mineral content of the soil, the microbial diversity is consequently affected.

## Figures and Tables

**Table 1:** Basic properties of the soil used in this study.

Soil Sample	K^+^ (mg/g)	Mg^2+^ (mg/g)	P (mg/g)	N (mg/g)	Si (mg/g)
S1	0.029 ± 0.007	0.006 ± 0.001	0.022 ± 0.002	5.366 ± 0.223	0.099 ± 0.007
S2	0.009 ± 0.002	0.044 ± 0.005	0.016 ± 0.004	3.805 ± 0.346	0.04 ± 0.012
S3	0.004 ± 0.002	0.056 ± 0.012	0.012 ± 0.006	5.476 ± 0.478	0.042 ± 0.005

S1: forest soil, S2: fertilized rubber tree rhizosphere soil from a rubber tree plantation, S3: fertilized bare soil from a plantation.

**Table 2:** Enumeration (bacterial number·g^−1^ soil) of total bacteria (TB) and potassium-solubilizing bacteria (KSB) from soils S1, S2, and S3, and the relative abundance of the cellulose-degrading bacteria of each soil.

Soil Sample	S1	S2	S3
TB	653	531	494
KSB	80	29	40
KSB/TB (%)	12.25%	5.46%	8.10%

**Table 3:** MiSeq sequencing results and diversity estimates for each sample.

Sample	Sequencing Results		Diversity Estimates			
	Reads	OTUs	ACE	Chao	Shannon	Simpson
**S1**	16,013	738	764	764	5.82	0.0054
**S2**	16,224	702	781	782	5.43	0.0097
**S3**	26,151	803	848	858	5.32	0.0151

The operational taxonomic units (OTUs) were defined with 97% similarity. The coverage percentages, richness estimators (abundance-based coverage estimator (ACE) and Chao), and diversity indices (Shannon and Simpson) were calculated.

**Table 4:** Number of isolates from samples S1, S2, and S3, classified by Gram typing, sequence analysis, and Amplified Ribosomal DNA Restriction Analysis (ARDRA) typing.

Gram	Genus	ARDRA	S1	S2	S3	Total
+	*Bacillus*	Group (I)				
		A	17	0	0	17
		B	8	0	0	8
		C	12	0	1	13
		E	14	0	0	14
		F	0	4	1	5
		G	2	8	0	10
		H	10	7	2	19
−	*Enterobacter*	Group (II)				
		I	0	3	0	3
		J	0	5	1	6
		K	0	1	7	8
		L	1	0	8	9
−	*Pseudomonas*	Group (III)				
		D	16	1	1	18
		N	0	0	17	17
−	*Achromobacter*	Group (IV)				
		M	0	0	2	2
		Total	80	29	40	149

S1: forest soil; S2: fertilized rubber tree rhizosphere soil from a rubber tree plantation; S3: fertilized bare soil from a plantation; +: Gram positive; −: Gram negative
